# School-Based Influenza Vaccination: Parents’ Perspectives

**DOI:** 10.1371/journal.pone.0093490

**Published:** 2014-03-31

**Authors:** Candace Lind, Margaret L. Russell, Judy MacDonald, Ramona Collins, Christine J. Frank, Amy E. Davis

**Affiliations:** 1 Faculty of Nursing, University of Calgary, Calgary, Alberta, Canada; 2 Department of Community Health Sciences, Faculty of Medicine, University of Calgary, Calgary, Alberta, Canada; 3 Alberta Health Services and Department of Community Health Sciences, Faculty of Medicine, University of Calgary, Calgary, Alberta, Canada; Public Health England, United Kingdom

## Abstract

**Background:**

School-age children are important drivers of annual influenza epidemics yet influenza vaccination coverage of this population is low despite universal publicly funded influenza vaccination in Alberta, Canada. Immunizing children at school may potentially increase vaccine uptake. As parents are a key stakeholder group for such a program, it is important to consider their concerns.

**Purpose:**

We explored parents’ perspectives on the acceptability of adding an annual influenza immunization to the immunization program that is currently delivered in Alberta schools, and obtained suggestions for structuring such a program.

**Participants:**

Forty-eight parents of children aged 5-18 years participated in 9 focus groups. Participants lived in urban areas of the Alberta Health Services Calgary Zone.

**Findings:**

Three major themes emerged: Advantages of school-based influenza vaccination (SBIV), Disadvantages of SBIV, and Implications for program design & delivery. Advantages were perceived to occur for different populations: children (e.g. emotional support), families (e.g. convenience), the community (e.g. benefits for school and multicultural communities), the health sector (e.g. reductions in costs due to burden of illness) and to society at large (e.g. indirect conduit of information about health services, building structure for pandemic preparedness, building healthy lifestyles). Disadvantages, however, might also occur for children (e.g. older children less likely to be immunized), families (e.g. communication challenges, perceived loss of parental control over information, choices and decisions) and the education sector (loss of instructional time). Nine second-level themes emerged within the major theme of Implications for program design & delivery: program goals/objectives, consent process, stakeholder consultation, age-appropriate program, education, communication, logistics, immunizing agent, and clinic process.

**Conclusions:**

Parents perceived advantages and disadvantages to delivering annual seasonal influenza immunizations to children at school. Their input gives a framework of issues to address in order to construct robust, acceptable programs for delivering influenza or other vaccines in schools.

## Introduction

Each year, thousands of persons in Canada and elsewhere miss school and work, visit emergency departments and physician offices, are hospitalized or die from influenza [1]–[9]. Elective surgeries have been cancelled in more than one province in Canada because staff have had to meet the care needs of surges in cases of seasonal influenza [10], [11]. Mathematical models and field research suggest that vaccinating school children provides indirect protection (herd immunity) to both household members and the community at large [12]–[16], [16]–[23] in addition to the direct benefits that individual children would accrue from annual vaccination. One strategy that is recommended to attain high levels of coverage for vaccines among children is to immunize them at school [24]. Immunizing children at school may also reduce disparities in vaccine coverage [25], [26]. In Canada, all 13 provinces and territories have used this strategy for decades to deliver vaccines recommended for school-age children [27]. All provinces and territories (P/T) publicly fund influenza vaccination: for targeted population groups in four P/T, and for the entire population (universal public funding) in nine [28]. Despite this, influenza vaccination coverage remains low, especially among children and youth [29], [30]. Delivering seasonal influenza vaccination to children at school is a strategy that is not widely used within Canadian provinces and has been tried (but not sustained) in only a few local areas within the province of Ontario [31]. While the perspectives of both health and education sector stakeholders must be considered in the design of such a program, parents are key stakeholders in any vaccination program that targets children as it is they who ultimately decide both if children should be vaccinated and where they should access this health service. If the views of these stakeholders are not considered, then a vaccination program that targets their children may fail. To gain insight into parents’ perspectives on adding school-based influenza vaccination (SBIV) to the school-based vaccination programs currently delivered within Canada, we studied parents in the Alberta Health Services Calgary Zone within the province of Alberta. In 2012, the population of this zone aged 5–18 years was 233,355. Of those, 36.7% were 5–9 years old, 34.1% 10–14 years old and 29.2% were 15–18 years old [32].

In Alberta, routine immunizations (including influenza) are publicly funded. Unlike many other Canadian P/T, childhood immunizations are provided exclusively by public health rather than through physician offices. The school immunization program is also exclusively provided by public health nurses and routinely vaccinates children in grades 5 and 9, but also provides immunizations at school in grade 1 for children who have not received recommended preschool immunizations [33]. Although physicians and pharmacists may deliver annual seasonal influenza vaccines to adults and older children, children under the age of nine years are immunized against influenza virtually exclusively by public health, primarily in public mass vaccination clinics [34], [35].

Since 2009, Alberta has recommended and publicly funded influenza vaccination for all persons aged 6 months or older. In October, 2012 intra-nasal (live attenuated) influenza vaccine began to be routinely offered to children 2–17 years old [36]. Children who have immune compromising conditions or severe asthma are offered inactivated influenza vaccine. Based upon Alberta Health Services Calgary Zone data [32], [37], 2012 influenza vaccination coverage for children aged 5–17 years was 15.6%.

## Research Design

### Ethics statement

The study was approved by the University of Calgary Conjoint Health Research Ethics Board (Ethics ID# 24083).

This study reports on the findings from the urban component of a larger research project encompassing parents living in urban and rural areas within 150 km of the City of Calgary, Alberta. This paper reports on the first phase of data collection, from urban parents from the City of Calgary participating in 9 face-to-face focus groups completed over an 8 month period of time.

Our primary objective was to gain insight into parents’ perspectives on adding school-based influenza vaccination to the school-based vaccination programs currently delivered within Alberta. A further intent of the project was to use the research results to inform public health policy and program managers about how SBIV programs need to be structured for success from the perspective of parents.

### Research team

Principal Investigator CL is a registered nurse with a background in public health nursing including experience in conducting school and mass vaccination immunization programs in Alberta. She holds a PhD and is an expert in qualitative methods. Co-Principal Investigator MLR is an epidemiologist (PhD) and medical specialist in Public Health and Preventive Medicine with experience and expertise in recruiting participants and conducting clinical trials and surveys. JM is a medical specialist in Public Health and Preventive Medicine and is responsible for communicable disease control (including immunization programs) in the Alberta Health Services Calgary Zone. Both MLR and JM have experience in planning and evaluating school-based vaccination and other vaccination programs within Alberta.

Hired as the project Research Associate, RC is a master of nursing graduate student who is a registered nurse with experience in community nursing and training in qualitative research. At the time of the study, Research Assistant CJF was an undergraduate nursing student with community health experience. Research Assistant AED has training and experience as a volunteer coordinator and in data analysis. Three authors acted as FG facilitators (RC, CL, MLR). CJF attended each FG, managed equipment and took field notes.

### Recruitment and sampling

Participants were recruited from the Calgary Zone of Alberta Health Services (AHS) with the assistance of several community agencies (school boards, health agencies, social service agencies, municipal departments) that had contact with parents throughout the Zone. The Calgary Zone is the most populous of the five health zones (36.7% of the population) of Alberta [38]. Advertising is a common recruitment strategy acceptable to the research ethics board. More direct recruitment, e.g., giving students an invitational letter at school to take home to their parents, was not feasible. The study was advertised using hard copy posters, links to electronic posters, social media and an advertisement in a family health magazine. Media interviews with the investigators also raised public awareness of the study. People who were interested in participation contacted the research team by telephone or electronic mail and were screened for eligibility based on responses to a self-administered questionnaire. It is possible that these parents were more interested in the topic of immunization than parents who did not respond to the advertisements. Eligible persons were parents of at least one child aged 5–18 years, who lived in a community within the AHS Calgary Zone, and were the main decision maker for their child’s health. We used stratified purposeful sampling [39] among eligible parents to attain maximum variation on having a child vaccinated against influenza and on being a lone parent. In addition to a $50 cash honorarium provided after focus group participation, those who had indicated during the screening process that they needed support for transportation to the focus group were provided with a transportation subsidy of up to $50, in an effort to facilitate the participation of a wide range of parents. However, only two parents required a transportation subsidy. Participants took part in only a single focus group and transcripts were not returned to participants for comment or editing.

### Methods

Focus groups (FG) provide an environment in which parents may feel safe to share beliefs that may differ from those of health professionals; can elicit information from the interactions among parents that parents may be hesitant to provide in individual interviews; and provide an opportunity for parents to interact with each other, build upon and clarify their opinions and elicit new ideas [39], [40]. They are considered to be particularly useful to gain lay perspectives on health service issues [41], thus were appropriate for attaining our objectives.

A trained facilitator used a semi-structured interview guide (Appendix S1) to explore parents’ experiences with having their children vaccinated at school. Additional questions included the advantages and disadvantages of SBIV; where parents would prefer to have their school-aged children vaccinated; under what circumstances would parents want to use or actually use a SBIV program; the features such a program should include; problems or issues that parents thought might arise with the addition of an annual influenza immunization to the vaccines currently delivered in their child’s school; and what would prevent parents from having their children participate in such a program.

We had originally planned to hold urban focus groups at community locations across the City of Calgary as we perceived that this would make them more geographically accessible to participants. However, as potential participants were widely scattered around the city, the University of Calgary proved to be a central, feasible and accessible (via car, bus or train) site for participants; thus all FG were held in a meeting room at the University of Calgary. FG lasted for a maximum of 90 minutes and were audio-taped and transcribed verbatim. The transcriptions and field notes were imported into data management software NVivo 10 (QSR International) for thematic analysis [42].

### Rigour

Rigour was addressed through careful attention to confirmability (by linking the findings and interpretations across data sources in readily discernable ways) [43], dependability (ensuring that the research was logical, traceable and documented) through regular team meeting discussions with documentation of decisions, and triangulation (multiple researchers reviewed the data analysis independently and then discussed together to clarify meanings and verify interpretations of the data) [42].

### Theoretic framework and Data analysis

The theoretical framework that guided the data analysis incorporated applied thematic analysis [42]. The transcripts were read and re-read by two investigators (MLR, CL) and research associate (RC) who developed a preliminary coding scheme after a detailed review of the data set. The data were coded using a combination of manual coding and coding with NVivo 10, with discussion of the coding decisions. Discrepancies were discussed and agreement using a consensus decision-making process was reached on an initial set of themes and sub-themes. The two investigators and the research associate coded the data and discussed the findings which resulted in revisions to the coding. To establish face validity, JM read the transcripts and critically reviewed the coding scheme.

### Data saturation

We recruited participants from a large urban area (Calgary) of the Alberta Health Services Calgary Zone and held focus groups until no new knowledge was obtained from new participants (i.e., data saturation was attained) based on the blind and independent judgement of two of the investigators (MLR, CL).

## Results

### Description of participants

Forty-eight parents of children aged 5–18 years participated in nine FG conducted between April and November of 2012. One of the 48 participants withdrew at the beginning of a FG because of a family emergency. Of the nine FG, six included only parents who had at least one child who had previously been immunized against influenza, two included only parents who had never had a child immunized for influenza, and one included both types of parents. Three of the nine FG were comprised exclusively of lone parents. In Alberta, grades Kindergarten (K)-6 are generally considered to be ‘elementary’ school, grades 7–9 ‘junior high school’, and grades 10–12 ‘high school’. All 9 FG included participants with children in grade level K-6. Four FG had participants with children across all three grade levels of school, one FG had participants with children only in K-6, two FG had participants with children in K-6 and grades 7–9, and two FG had participants with children in K-6 and grades 10–12. Demographic characteristics of participants are described in Table 1. Most had been immunized against influenza on at least one occasion, although during their FG some self-identified as vaccine hesitant.

**Table 1:** Description of participants.

	PARTICIPANTS (N = 48)	
	N	%
GENDER		
Male	9	18.8
Female	39	81.2
AGE-GROUP		
20–39 years	17	35.4
40 years or older	30	62.5
Data not provided	1	2.1
LONE PARENT		
Yes	9	18.8
No	38	79.2
Data not provided	1	2.1
HIGHEST LEVEL OF EDUCATION ATTAINED		
High school or less	3	6.3
Some post-secondary	4	8.3
Post-secondary certificate or trades certificate	12	25.0
University degree	29	60.4
NUMBER OF CHILDREN IN FAMILY		
1	12	25.0
2	22	45.8
3	11	22.9
4	3	6.3
Median number of children in family	2.0	___
REQUIRED TRANSPORTATION SUBSIDY TO ATTEND FOCUS GROUP		
Yes	2	4.2
No	46	95.8
PARTICIPANT EVER VACCINATED AGAINST INFLUENZA		
Yes	40	83.3
No	6	12.5
Not sure/missing	2	4.2
AT LEAST ONE CHILD EVER VACCINATED AGAINST INFLUENZA		
Yes	39	81.3
No	9	18.8

### Data organizing tool

As data were analyzed it became apparent there were multiple levels of action and recommendations for strategies for building effective SBIV that were arising from the data. The population health promotion model (PHPM) [44] was chosen as a tool to assist in organizing the emerging themes. This model provides a guide to addressing actions to improve health. It addresses ‘with whom’ we should take action (i.e., at which population level ranging from individual to society as a whole), types of action strategies (e.g., re-orienting health services, building healthy environments) and what determinants of health to take action upon (including health services, personal health practices and coping skills). The PHPM provided a guide for organizing and understanding parents’ views of SBIV, for understanding parents’ recommendations for action across multiple levels to build an effective school-based influenza vaccination strategy and for framing strategies for improving influenza vaccination uptake for school-aged children in Alberta.

### Emergent themes

Three broad first level themes emerged: Advantages (of SBIV), Disadvantages (of SBIV), and Implications for program design and delivery (Tables 2, 3, 4). Under each of the first level themes of Advantages and Disadvantages we categorized second level data themes according to the PHPM levels where action can be taken. These were labelled Child (representing the individual level), Family, Community, Sector, and Society. Under the Implications for program design and delivery first level theme, second level themes were conceptually linked to the PHPM action strategy of ‘reorienting health services’. Examples of these include education, communication and logistics. A third level of headings (another layer of sub-themes) represented unique attributes under each of the second level themes.

**Table 2:** Main findings: Advantages (Level 1 Theme).

Level 2 Theme	Level 3 Theme	Level 4 Theme
Child	Emotional support for immunization process	Desensitization to being vaccinated
		Peer support
	Health	Building healthy lifestyles
Family	Convenience	
	Promoting health of families	
Community	Benefits for school community	
	Benefits for multicultural communities	
Sector	Health sector benefits	Financial & resource benefits
Society	Pandemic preparation	
	Financial	Reduction in societal costs from preventable illness
	Health	Building healthy lifestyle
		Herd immunity

**Table 3:** Main findings: Disadvantages (Level 1 Theme).

Level 2 Theme	Level 3 theme	Level 4 theme
Child	Health	Older children not immunized
Family	Communication challenges	
	Perceived challenges to parental control	
	Unacceptability of the consequences of not participating in the program	
	Health	Parents not immunized
Sector	Education sector risks	Loss of instructional time

**Table 4:** Main findings: Implications for Program Design & Delivery (Level 1 Theme).

Level 2 Theme	Level 3 theme	Level 4 theme
Program Goal/objectives		
Consent process	Voluntary program	
Stakeholder consultation		
Age appropriate program		
Education	Message content	
	Education strategies	
Communication	Multiple channels	
	Multiple languages	
	Multiple sources	
	Credible sources	
Logistics	Timing	
	Clinic space	
	Staffing	Staff training
	Roles of health vs. education sector	
Immunizing Agent		
Clinic process	Observation for adverse reactions following immunization	
	Fear management strategies	
	Incentives & rewards	

### Advantages (Table 2)

Advantages of SBIV from parents’ perspectives occur at the levels of child, family, community, health sector and society.

#### Child

There were two third level themes under the heading Child: Emotional (emotional support provided for the vaccination process) and Health.

The theme Emotional captured two concepts: the importance of desensitizing the vaccination experience for children, and the value of peer support. Having an annual program in schools would help desensitize children by normalizing the immunization experience and reducing their anxiety and fear related to vaccination: “…I have seen some pretty big kids become puddles, crying and crying and talking through it, as well as my daughter, right. So I think that if you had an opportunity to have the flu shot every year and some kids did it then that would maybe take the edge off of all that anxiety if it was more of a commonplace [experience]…”(FG 4). A group immunization experience may provide peer support for children, and help to make vaccination more of a positive experience. “My daughter is currently 13 …and she has always actually liked that the [other] vaccinations were in school because then all of the children got them at the same time and they all felt comfortable that they were going through the same pain at the same time so they all really kind of confided in each other, comforted each other I guess, so they really enjoyed that.” (FG 6).

Advantages of SBIV at the Child level also included a third level theme, Health. An advantage of SBIV included an opportunity to incorporate further health teaching for children as immunization is only one component of building healthy lifestyles: “…don’t just push the vaccine… I like the idea of having it as a comprehensive thing like let’s talk about diet, exercise and vitamins and health habits. Handwashing 101 it needs to be reviewed repeatedly…” (FG 4).

#### Family

Advantages of SBIV to families encompassed two third-level themes: Convenience, and Promoting health of families.

Convenience was an important advantage heard across all nine focus groups, with the explanation that SBIV would offer parents reductions in time and effort to coordinate family activities to get to appointments: “… it comes down to time… I think a lot of parents have their children in activities in the evening and so it’s difficult for parents to find that time to bring their children to those [public health mass influenza vaccination] clinics… wait ¾ hours… [so]… I think that would alleviate that pressure on families…” (FG 6). For families with more than one child, particularly if public transportation had to be used, this was especially important: “… having school vaccinations also eases a burden on the parents that want to vaccinate their children but are really not able to get to the [clinic during clinic] hours… or they have to depend on a ride…” (FG 2). Comments about convenience addressed reductions in time demands on parents and children, including parents having to take time off work due to child illness or to take children to vaccination appointments: “Even for a two-parent household having a child home [ill with the flu]… for five days could really cause a lot of disruption, and if you have multiple children then you’re still having to deal with… trying to get them to school and back and forth.” (FG 3); “…when you look at [it] in light of the line ups and the time off work and days out of school trying to line up to get an immunization … then I would rather take that little bit of time out of school [for children to be immunized in school] …” (FG 4). Convenience also included that most families live closer to their respective schools than to a community health clinic.

Promoting the health of families was another theme that emerged. Parents believed it would be less likely that the children who participated in a vaccination program at school would infect other family members by bringing a virus home. Additionally, other family members (particularly parents) might be more likely to be vaccinated themselves as it would be easier to attend a mass vaccination clinic if not accompanied by children.

#### Community

At the Community level, there were perceived benefits for two distinct communities: the school community (students and staff of schools), and multi-cultural communities. The school community would benefit from a decrease in lost time and absences due to illness: “… if you come down with the flu… Kids are missing three, four, five days out of the week regardless… So if they’re going to take one day [for influenza immunization at school] so they’re not sick throughout the rest of the year then I think I would rather have [just that] one day lost.” (FG 1).

Multicultural communities may include people who face language or cultural barriers because of immigration and lack of familiarity with the healthcare system. These communities would indirectly benefit from SBIV through diffusion of information from the parents and children of their community who participate in an immunization program at school: “… [In] my community… there is a lot of people… who can’t even speak English. So they don’t know about the clinics. They don’t know what they need to do… They can have other people in the family tell them what’s going on and then they can make an informed decision without the language barrier, knowing that in the school this is going to happen…” (FG 1); “That’s got to be a huge plus for them, for their families, for their extended families and … consequently for … society.” (FG 2).

#### Health sector

Participants perceived that SBIV could provide financial advantages for the health sector as it would prevent some of the cost burden of treating preventable illness: “… it will cost more to treat your child for the flu than it is to vaccinate that child for many years while he is at school… Because if you can prevent one person from getting sick and going through the health care system … by vaccinating, then I think that outweighs the risk of the potential cost that it would be to vaccinate that many children every year.” (FG 2).

Some participants felt delivering influenza vaccine in schools would be a more efficient use of the time of public health nurses as compared to services delivered via a public health mass vaccination clinic: “I think the best way is to be done at the school, just to save resources… they [nurses] can just go into the school on certain days and get it done…” (FG 6).

#### Society

Extending beyond the levels of individuals, families, communities or sectors, other societal benefits would also occur. These would include benefits in the areas of better pandemic preparation, reductions in societal costs of illness, herd immunity and in building healthy habits that could be passed on to future generations for the benefit of society. Participants thought that vaccinating children against influenza at school would help with pandemic preparation by getting them accustomed to the idea of immunization and even by serving as a ‘pre-testing’ of pandemic roll-out plans: “I think it’s a great habit to get into because eventually the pandemic is going to come… It will hit the teenagers and the young people…” (FG 2); “… this [vaccinating for influenza at school] is the start of preventing worse things by having the habit [of young people being immunized]… set up… ready to go… It’s like getting your survival guide up and ready…” (FG 2).

Parents recognized that another societal benefit would include herd immunity (the interruption of transmission to unvaccinated people): “…the less people that you could possibly get the flu from. I mean, if there are more people that are immunized against it, overall there is going to be a healthier population…” (FG 7).

Societal costs of influenza illness would also be reduced: “… during flu season… the parents don’t get [the flu] … [because their children were vaccinated at school and don’t bring the virus home]… it would save so much money and resources for employers as well as parents that have children.” (FG 6).

Society would also benefit in other ways: “[children who are immunized against influenza in school are] … learning a good habit and … if that child eventually becomes a parent the chances of them giving vaccines to their kid is way higher… that goes on to build society quite a bit in the future.” (FG 2).

### Disadvantages (Table 3)

Parents’ perceptions of the disadvantages of SBIV are organized under the second level theme headings of Child, Family and Education sector.

#### Child

A disadvantage of vaccinating children against influenza at school would be the potential that older children might not get immunized against influenza: “The older kids probably just wouldn’t go. In high school [if I was a student] I would be like - oh is something going on? Free period. Off I go -… but [in] junior high you can’t really take off.” (FG 1).

#### Family

Perceived disadvantages at the family level were related to communication challenges, perceived challenges to parental control, the unacceptability of the consequences of not participating in a program, and negative impacts on the health of parents.

Communication challenges included comments about past experiences with children being immunized through the existing public health school immunization program: “I didn’t feel that they communicated with me. Like there was a series of shots for my daughter and they told me when they got the first one and then they’re supposed to get the second one and it was delayed. They eventually got it. But it wasn’t communicated really well to me when the subsequent shots took place…” (FG 1). There was also a concern about a lack of advanced notice: “It was too much for me all at once. I didn’t have enough knowledge to go ahead and say ‘yes’ because it seems we’re informed and the shots were right there…” (FG 1).

The types of challenge may vary with the grade level of the student: “… I think you are supposed to get something from the nurse that is stamped and initialed that they did get the shot. … But then with the junior high/high school kid [I asked] ‘did you get the paper?’ ‘Yeah. It’s in my locker.’ You know? So how do you really know they did or didn’t [get it]?” [parent 1], “…Well that could be an e-mail sent out” [parent 2], “Then you don’t have any proof [that the child had actually received the vaccine]. I mean an e-mail can be faked…” [parent 3] (FG 1).

The third level theme of Perceived challenges to parental control captures parents’ desire to control information, choices and decisions offered to their children that are in conflict with those parents’ beliefs and values: “So you hear the words ‘everybody has to have a flu shot’ and you think well I’m not okay with that. You’re not telling me what to do.” (FG 2); “If parents are strong against the flu shot… kids [may challenge parents, saying]… ‘teachers told us it is good to have the flu shot and you said no’…” (FG 7).

Concerns about the consequences of not participating in an influenza immunization program that was offered at school are represented by the third level theme of Unacceptability of the consequences of not participating in a program. There were perceptions that parents might feel pressured into having their children immunized for fear of being labelled a bad parent: “… if I don’t do [it] then I’m going to look like I’m the bad parent…” (FG 1); “… you run the risk of being the parent who chose not to do something and then … that kid causes an outbreak in school…because you opted out.” (FG 3).

There were concerns that in a school setting it is not possible to maintain privacy about parental decisions: “… if you did opt out everybody knows, even though they try to maintain privacy, because your kid will talk and say – ‘oh I didn’t have to do it because my mother said no’…” (FG 3). Because of this lack of privacy, parents raised concerns that these children would feel singled out and might be picked on by their peers: “…you run the risk of kids being singled out like ‘you’re going to get cooties’ or ‘you’re going to [get] sick because you didn’t get the shot’… [This constitutes] ammunition or fodder for kids to pick on each other…” (FG 2).

The final third level theme under Family was Negative impacts on the health of parents. Having children immunized against influenza in school could result in fewer parents being vaccinated: “… it might prevent the parents from getting it because, right now… the parents … make sure the whole family is vaccinated but [if the children were vaccinated at school] it would just be the kids… I would be all ‘oh I’ll get to it’ but I never do…” (FG 1).

#### Education sector

Disadvantages (e.g., costs) for schools could include loss of instructional time and disruptions to school staff caused by an immunization program: “… there is the cost … of the children being pulled out of class time, the cost associated with having all the staff, teachers, admin staff whatever rounding up the kids…, the discussions around it; … that is going to take away from a little bit of class time - with the anxiety levels for different children and stuff, so I mean there would be a minor cost associated with and a little bit of disruption, [including] the paperwork, managing it…” (FG 4).

### Implications for Program Design & Delivery (Table 4)

The final first level theme Implications for program design & delivery arose as a strong focus of participant discussions, with many suggestions arising from interactions where parents brainstormed and built upon viewpoints of each other about how a SBIV program should be constructed. Nine level two themes emerged under this final first level theme’s section. These included: Program goal/objectives, Consent process, Stakeholder consultation, Age appropriate program, Education, Communication, Logistics, Immunizing agent, and Clinic process.

In addition, participants suggested program planners should model SBIV on other school vaccination programs that were successful: “I think modelling it after programs that have been effective like the HPV or the Grade 5 [hepatitis B program]; it’s a good system how they do it all, so kind of role modelling it after one.” (FG 5). Parents also thought it would be important to pilot the program or roll it out gradually to assess local acceptance: “Start it off in one section, say one school… test it out, see how it works and how it goes. Learn from that… From there expand it a little bit to maybe a couple of more schools, and work again from there.” (FG 9).

#### Program goal/objectives

Clear goals of a SBIV must be developed to drive implementation decisions and facilitate communication with others. In addition, parents commented that evidence in support of the goals would be required.

Suggestions for program goals were made. The goal of a program might be to reduce influenza transmission, indirectly protecting others: “… it’s not school-aged kids who suffer the most [from influenza]. It’s like little Tommy who has the sniffles and goes to visit Grandma, and Grandma comes down with something more serious. So sure we need to break the chain of transmission there…” (FG 2). Another program goal might be to educate and empower children and others for the future: “… it is almost an education part of our school system for bringing it into the schools to empower the kids in the schools too.” (FG 1).

Participants suggested the general population could be given access to the program: on the days that nurses come to school to immunize children during class time, they should include an evening or after-school continuation of the clinic to permit parents or other nearby community residents to be immunized: “You could add onto that - people in the area.” [parent 1], “That way then you could also get your residential people who might not have gone out to a flu clinic because it is inconvenient, they couldn’t make an appointment or [go to] their drug store or whatever. It’s close to them and they could just pop in and get it done.” [parent 2] (FG 1). If the general population was given access to the program, then a benefit from this might be a contribution to other goals, such as building community: “… even the family of preschoolers [if] they have a chance to go, have a chance to get a vaccination at school, it has an opportunity to bring the community together … we really [have to] physically come together and we have to chat with each other and bring a sense of belonging to this community.” (FG 2).

Overlapping with the Advantages theme, the program could be used to promote positive attitudes to vaccination, thus benefitting society: “… in our society there is a negative connotation to vaccines… [however] if in school you’re constantly told take this, it is good for you… it is almost a [positive] desensitization process…” (FG 2).

#### Consent process

Parents agreed that an SBIV program must be voluntary and the informed consent process would have to include information on both the benefits of and potential harms associated with vaccines. Parents suggested that this does not always occur: “I think in general that the information that is provided tends to be very one-sided and very pro-immunizations…” (FG 4).

Consent would have to be an ‘opt in’ process (i.e., parents would have to consent each time to their child’s participation), rather than an ‘opt-out’ one (where consent is assumed unless parents explicitly refuse permission). Parents also suggested that consent might be given through the use of electronic means in addition to paper forms.

#### Stakeholder consultation

Parents thought there might be a need to get input from schools and parents, and suggested strategies for obtaining this input: “… survey… to see who will actually use this system… do we have enough users in these neighbourhoods in the city… and in these particular schools [so here] this is a good idea but in… schools over here it’s not a good idea; it’s not flying with this group of parents for whatever reason…” (FG 3).

#### Age-appropriate program

The operational features of the program should depend on the ages of the children being targeted, as children’s needs vary with their age and grade levels: “I think the staff who gives it needs, especially for the younger kids, … to be compassionate and have a great rapport with little kids and kids in general; whereas maybe you could be pretty much black and white and straightforward with the older kids like [in] the Junior High and the Senior High [schools].” (FG 5). Suggestions for making the program more acceptable for younger children would include allowing parents to accompany their child to a SBIV. Parents also proposed that older children might be offered an option: “…You either do it at the school or you do it with Mum and Dad at the clinic…” (FG 6).

#### Education

Education was perceived to be a critical component of program design. Parents made suggestions for target audiences for education, for content of educational messages and for educational strategies that could be used. Target audiences should include parents, children and teachers.

Recommended content would include information about the disease: “… how people can get really, really sick with the flu and further complications…” (FG 1). Parents would also need education on the rationale for the program, including cost-benefit, and the cost to parents. Parents wanted more information about the vaccine: “… What was it grown in and all that stuff…” (FG 2); but also thought that education content should cover more than just vaccine and immunization information: “… you still need to wash your hands and blow your nose and put your tissue in the garbage can and cough into your elbow not into your hands…” (FG 3).

Educational content would need to address the concerns that parents expressed about potential harms from influenza vaccines and perceived unfavourable benefit-risk ratios for annual influenza immunization. Some parents thought the risks of the vaccination outweighed the benefits for healthy children: “My son hasn’t had the flu… he’s made it through six years, I don’t want to start giving him shots every year for something he’s never going to get… [it’s] not a completely risk-free immunization…” (FG 3). Another parent was concerned about long term cumulative harmful effects of vaccines on children’s bodies. Many parents viewed the seasonal influenza as a mild ailment in otherwise healthy children, and hence not serious enough to warrant vaccination: “It’s just the flu!” (FG 9). As there are multiple strains of the virus, some parents were also not convinced a vaccination would protect their children “… the flu shot protects against certain strains and not every single strain. So it doesn’t mean that little Sally isn’t going to get the flu…” (FG 9).

An important strategy for improving children’s education would be embedding vaccinations in the school curriculum: “… they could even have a day in the curriculum about vaccines, why people do it, why countries do it and just a little bit of background so that they would understand it a bit more, sort of build it into the curriculum…” (FG 5). An advantage is that this could also be a strategy to indirectly educate parents: “… I do learn a lot of things from my children when they come home and say ‘well this is what we did today’…” (FG 2).

#### Communication

Communication would be a critical program component. Parents wanted the communication to not only include soliciting consents from parents, but to also ensure records and information would return home to the parents: “… you want an official thing to put in their record book…” (FG 1). Parents indicated a need to use multiple channels (paper, electronic, media) to deliver messages about the program, particularly because a reliance on having children transport hard copy documents including vaccination records to and from parents (‘back-pack’ delivery) was not appropriate for all age groups: “… the tricky part, as they got a little bit older, [was] they didn’t bring the form back home…” (FG 2). Some suggested how to use electronic media: “A document can be sent in a pdf format that can be printed off. Sign it off and bring it to school…” (FG 2). Social media, ad campaigns and public service announcements were also suggested communication strategies.

Consistent messages (provided in multiple languages) should come from multiple sources (not just the school or health agencies). This would be particularly important to reach parents or other members of a community such as new immigrants or those facing language or cultural barriers: “… spread the word in government agencies [that we need better messaging], we will be very appreciative…” (FG 2); “… those that are newcomers … this may be their first brush-up against vaccination…” (FG 2).

Messages and information must come from credible sources and must take into account that parents vary in their perceptions of what counts as a credible source: “I need more information and I don’t know where to get it because right now it’s all from the same quasi-reputable [source, and] … I needed a broader sampling of this data to show up across multiple medias before I could feel comfortable that I was well enough educated.” (FG 3).

#### Logistics

Program logistics include issues about the timing, location (clinic space), staffing and training related to planning and delivering influenza vaccination at school. Timing included time of year, time of day and lead time needed for various program components. Parents questioned if the program would be offered at the same times of the year as key school activities (e.g., teacher professional development days)? Would it only be offered during regular school hours, would there be sufficient lead time so teachers and school administrators could be prepared? Would parents have enough time to make an informed decision about program participation, and how could parents who did not yet have school-aged children become aware that this would be a possible part of their child’s experiences at school?

Some parents suggested a process that would use “… a number of nurses at the same time on the same day in an environment like the school gym and then having different stations in the gym for multiple students to get vaccinated at the same time, maybe a class at a time.” (FG 6). Clinic space should include a privacy room to prevent embarrassment for those who had to partially disrobe for an injection, or for children who were distressed: “… set aside … quiet areas or a private area for children of maybe different nationalities that can’t show their arms… or [for] those kids that are traumatized…” (FG 1).

Some parents were concerned that clinic staff might not be appropriately qualified health professionals, indicating a need for specific information from program delivery staff, and perhaps a generalized lack of awareness of the qualifications of staff delivering current immunizations: “Are they qualified to do this? Or are they just some stranger randomly picked off the street [?]…” (FG 9); “… they need to provide a … nurse at the school. I don't think it’s fair to put that onus on a teacher…” (FG 8); “…Would you be guaranteed that this is going to be a nurse doing it?… anybody could give the nasal [spray] so is it going to be anybody giving that nasal [vaccine]?” (FG 9). Others recognized that a qualified health professional would be required to immunize: “… registered nurses are more educated in regards to looking at big picture reaction for allergies, anaphylactic reaction those sorts of things. I would have that preference for an RN to give my child the injection.” (FG 6). Parents were also concerned that clinic staff must be adequately trained in procedures to ensure that children were not accidentally immunized without parental consent or were immunized more than once in error. Overlapping with education needs, showing a lack of knowledge of even basic safety procedures or infection control procedures, some parents suggested: “… make sure they … [are] using disposable syringes or if … not disposable…properly sterilizing [equipment]…” (FG 6).

Finally, parents wondered about the different roles of the school and health systems that were providing the immunizations in schools, related to the logistics of managing the paperwork for the program. A number of parents did not appear to understand that vaccines were delivered by the health sector; instead they thought the education sector delivered vaccinations and therefore SBIV might take needed resources (including money) away from schools.

#### Immunizing agent

Parents perceived that an influenza vaccine that could be given in a form other than injection would be a good idea and some mentioned reductions in risks of infection or a sore arm, and greater comfort for those afraid of needles (especially younger children). However, others mentioned the possibility of a sore nose from nasal sprays or thought that vaccines delivered nasally might not be acceptable: “My kids would be weirded out by the noses …. because we’ve never done anything to [their] nose[s]…” (FG 1).

#### Clinic process

The Clinic process second level theme included strategies to manage fear, monitoring for adverse reactions, and the use of incentives and rewards. Although all participants had one or more school-age children, many commented that their children were too young for either the parent or child to have had experience with Alberta’s school immunization programs, which start in grade 5.

Overlapping with the Advantages theme section, parents thought younger children might be most likely to be fearful, but on clinic day children of all ages would benefit from the support of their peers during immunizations at school. However, they also felt younger children in particular might require other strategies to reduce fear, anxiety and pain related to immunization. Suggestions for the younger children (which overlapped with theme Age appropriate program) included: “… for the elementary kids as well… it would be good to have lots of volunteers to assist, especially … in kindergarten and Grade 1…” (FG 4); “… [offer] a numbing cream…”; “for parents who don’t like needles… [offer] the nasal spray.” (FG 5); “… [if] parents can be there for their child’s fear… Tylenol can be given as well…” (FG 8); and “… a notice could go out saying shots tomorrow, kids can feel free to bring their comfort toy” (FG 1). There were many potential roles for parents and the school staff in managing fear. The teacher could accompany the class to the immunization clinic as part of a calm, normal school day process; and the teacher could provide a trusted, familiar face that might be reassuring to young children.

As is current practice, children would need to be monitored for adverse reactions: “… the mass flu shots [clinic] … had a station for people who are having bad reactions… so they would have to have some sort of provision like that at the school…” (FG 5).

Incentives and rewards for being immunized should be considered for children: “… especially for the little ones when they finish their flu shot in the clinic they get a sticker or some reward thing to make them feel good…” (FG 6). For high-school children, parents suggested having food available might make the immunization process more attractive. However, parents also expressed concern that if a classroom reward was given only to those who had been immunized, children who were not immunized would feel left out; thus rewards given in the classroom should be given to all children on vaccination day.

## Discussion

Several studies of school delivery of influenza vaccine in the United States have been published [45]–[47]; however the structure and funding of immunization programs and health care services generally is substantially different from that in Canada. This paper adds to the literature on the annual delivery of seasonal influenza vaccine to children at school, by expanding the contexts in which the issue is considered and by using the PHPM framework to organize findings with respect to populations, types of action strategies and determinants of health upon which to take action.

The PHPM assisted in organizing the perceived advantages and disadvantages of adding SBIV to currently delivered immunization programs in Alberta schools. Parents perceived advantages would occur for populations at the levels of the child, family, community, health sector and society in general. For children, a particular advantage would be receiving emotional support from peers during the immunization process; depending on how the program was ultimately structured, another advantage for children would be an addition to the development of healthy lifestyles. The primary advantage for families was convenience although parents recognized potential health benefits for families included the reduced risk of an immunized child introducing infection into the household. Parents gave many examples of being burdened with trying to schedule influenza vaccinations within other competing family needs. Many variables contributed to their sense of burden: the stressful nature of having to prioritize and juggle activities after school, having more than one child to attend to, living as a lone parent family, work constraints, and lack of a vehicle. Although participants used the word ‘convenience’, their comments addressed issues that have been found by others to be barriers to immunization against influenza and other vaccine preventable diseases in diverse settings [48]–[50]. Many of these barriers relate to two of the five dimensions of access to health services: accommodation – the extent to which the providers’ operations are organized to meet the constraints of the user, and geographic accessibility [51].

For community level advantages, parents perceived that school communities (students and staff of schools) would incur less absence due to illness, while multicultural communities would benefit through the diffusion of information from parents and children who attend schools in which the SBIV program is offered. A similar strategy has been assessed and found to have value for hypertension education in a general population [52], [53]. Participants also thought there would be benefits to the health sector in terms of reduced costs from treating preventable illness. Finally, society as a whole would benefit with better pandemic preparation, reductions in societal costs of illness, development of herd immunity and building healthy habits in individuals and families that could be passed across generations. It is interesting that our participants identified herd immunity as a benefit. A systematic review of the literature found that although parents recognized and valued the concept of herd immunity for other vaccines, it did not ‘resonate’ with parents who participated in focus groups about influenza immunization [54].

Disadvantages were perceived to potentially affect fewer populations: the child, family and education sector. Older children might be less likely to attend school vaccination clinics than younger children, so (in the absence of other strategies for immunization) may not be immunized against influenza. Perceived challenges to parental control and the unacceptability of the consequences of not participating in a program were also raised, as was the possibility that parents might be less likely to be immunized against influenza because they would be left out of an SBIV program that did not involve all family members attending immunization clinics together. Some parents described acting as role models during family outings to get immunized, and this role modelling would be lost if families did not seek immunization as a family. The issue of parental control has been identified elsewhere [55] in the context of adolescents making vaccination decisions without parental consent.

Strategies to address challenges to parental control that should be considered in program design would include ensuring respect for parent choice to opt out, and that children not vaccinated would still receive the same rewards as their fellow students on vaccination day, so they would not feel penalized for their parents’ decisions. Prior literature has focused only on rewards for returning consent forms [56].

Communication and education for both themselves and their children were viewed by parents as important components of SBIV with implications for program design and delivery. Education should include credible information about influenza, risks and benefits of vaccination, vaccine ingredients, financial costs, and program goals and objectives. We were surprised that some participants were concerned that vaccinators might be just people ‘taken off the street’ and that needles might not be sterile. Education thus should also include information about the training and qualifications of personnel who vaccinate; concerns about this have been identified among a minority of parents elsewhere [50]. As many parents had concerns and misperceptions about the aforementioned, increased education is necessary to help parents make informed decisions. As one parent mentioned that vaccinations in general have a negative connotation in society, providing educational opportunities for parents may acknowledge parents’ concerns and build awareness of the benefits of influenza vaccinations. Parents gave many suggestions for communication strategies to meet the needs of all parents, including families who face language and cultural barriers.

Using age appropriate educational strategies, children should learn about the benefits of vaccination in the context of health promotion and disease prevention for themselves, their families, and society. Children would be provided with a solid foundation upon which to build critical thinking skills, essential for making informed decisions about health promotion as they navigate from childhood to adulthood. There is a substantial need for marketing and education about influenza vaccination at multiple levels including child, family, community and society. Participants suggested there is insufficient information available to the public with explanations of the importance of influenza immunization, explanations of the components of the vaccine, its safety, and even basic information such as sterility of equipment and the qualifications of vaccine providers. Multiple myths abound about vaccination in general and influenza in particular [57], [58], which need to be counter-balanced by scientific evidence, facts and better marketing. Parents asked what is accurate information and where do we get it? Vaccine hesitancy generally is a complex phenomenon, and approaches to communication need to address not only common concerns but also use evidence based strategies to address them, including strategies to interpret and evaluate information found on the Internet [59] and appropriate techniques to use for debunking vaccination myths [60]–[63].

Addressing children’s fears of vaccinations was viewed as an important consideration with implications for program design and delivery. Fear was viewed as a natural response to vaccinations, especially in younger children. Therefore, many age-appropriate strategies were recommended.

Many of our findings are not unique. Middleman and colleagues [47] used FG to examine factors that might influence parent decisions to have their children participate in school-located influenza vaccination programs. Similar to our findings, their findings included such benefits as convenience and stopping the spread of illness. Factors that would make parents more likely to have their children vaccinated at school included convenience, free or low cost vaccine, and knowing the credentials of the vaccinator. Their finding of the importance of free or low cost vaccine may have reflected an environment in which influenza immunization was not universally publicly funded. The potential for children to offer emotional support to each other, and the opportunity of SBIV to build healthy habits were not mentioned in their research. ‘Drawbacks’ included side-effects from vaccination, parents not being around for vaccination, scheduling difficulties for schools and children afraid to get vaccinated in front of peers. It is interesting that their research findings did not include family disadvantages such as perceived challenges to parental control. Similar to our findings, factors that would make parents less likely to have their children vaccinated at school included not having good information, concerns about the credentials of vaccinators and concerns about sanitation and schools being able to handle side-effects of immunization. A systematic review of the literature identified several best practices for implementing influenza vaccination in schools [56], including education of parents and students about the disease, benefits and side-effects of immunization; involving and considering the concerns of stakeholders such as schools and teachers; and communication in multiple languages.

Second level themes within Program design & delivery ranged from high level programmatic considerations to considerations for detailed program delivery. There were similarities between many of these second level themes and many of the important questions and issues that are often recommended to be addressed in program evaluations. Perhaps this is not surprising as the best program design should also consider program evaluation. Questions such as “what is the program and in what context does it exist?” and addressing program descriptions that convey the mission and objectives of the program in sufficient detail to ensure that program goals and strategies are understood [64] are common to both.

Our findings indicate that one cannot assume that parents have even basic information on vaccine delivery, what constitutes a vaccine, how vaccines are delivered and by whom, safety and other issues; or how the health and education sectors collaborate in the delivery of current school-based vaccine programs. We found that although overall our focus group participants were well educated, parents were quite variable in their knowledge. Thus, regardless of parents’ educational attainment these basic questions must be addressed in marketing influenza immunizations. Campaigns that just focus on a message that it is flu season and people should get vaccinated do not address the underlying issues that prevent a number of parents from having their children immunized. We speculate that vaccinating children at school, compared to other strategies for delivering influenza vaccine to children, may result in increased vaccine uptake providing the program is designed to address issues raised by our participants, including substantial communication and education efforts tailored to local parents’ needs, questions or concerns. Vaccine cost is an important program component (even if other elements of the program are optimally designed) [47], particularly for low income populations. In the United States a two year study to compare the efficacy of a school-based or provider-based multi-component (education, free vaccination) intervention with a standard-of-care approach was conducted in an area with a substantial proportion of low-income families [65]. By the second year of the study, the school-based intervention had demonstrated significantly higher vaccination uptake than the provider-based intervention (delivered by physicians and county health departments). There was a change from a baseline of 5.2% of students vaccinated to 30.4% of students vaccinated in the school-based intervention. In contrast, among those allocated to the provider-based intervention the observed change in vaccine uptake was from 10% at baseline to 18.4%. Students were more than twice as likely to be vaccinated for influenza in the school intervention than students in the standard-of-care county [65]. In the province of Ontario, Canada where publicly funded influenza immunization has been available to all since 2000, some public health units (PHU) used SBIV. Statistically significant, (modestly) larger proportions of children who lived in PHU that used SBIV were vaccinated against influenza than those in PHU that did not use SBIV (36% vs. 24% for those aged 4-11 years; 39% vs. 30% for those aged 12-19 years) [31].

### Strengths & limitations

The strengths of this study include careful attention to rigour and inclusion of participants who had differing levels of experiences with influenza immunization for themselves and their children, including some parents who self-identified as vaccine hesitant and some who volunteered information during focus groups that they did not choose to vaccinate their children against any vaccine-preventable disease. Additional strengths were the inclusion of parents of children across all grade levels, and parents of varying marital status. Although we had some participants with low educational attainment and possibly also low income (using self-identified need for transportation subsidy for FG attendance as an indicator of low income), it is possible that we may not have fully captured the range of concerns and opinions of such groups if they differed from those of other higher education or income groups. Limitations include that the results of this study cannot be generalized to other populations due to the nature and intent of the research design. Qualitative research is not intended to provide statistically generalizable estimates to a larger population. Rather, others should assess the transferability and usefulness of the research findings for their own practice contexts.

## Conclusion

All school immunization programs must pay careful attention to design and implementation if programs are to be optimized for success and sustainability. Consultation with key stakeholders (including parents) and consultation to tailor SBIV to a local school’s context may be optimal.

## Supporting Information

Appendix S1 **Semi-structured interview guide.**
(DOCX)
