# Supplementary material for: School-Based Influenza Vaccination: Parents’ Perspectives
Source: PLoS One. 2014 Mar 31;9(3):e93490. doi: 10.1371/journal.pone.0093490 (PMC3970961; doi:10.1371/journal.pone.0093490)
Supplement: Appendix S1 — Semi-structured interview guide. (DOCX) [file pone.0093490.s001.docx]

**Appendix S1: Semi-structured interview guide**

Welcome everyone and thank you for agreeing to meet with us. My name is ______________. As you know, we are interested in getting opinions from parents of school-aged children about school-based vaccination programs, in particular about adding a yearly flu vaccination to the vaccination programs already delivered in schools. To clarify, this is NOT a discussion about whether or not in general children should be vaccinated; and it is not a discussion about whether vaccines are a good thing or a bad thing, or about how vaccines are made. It is a discussion about the advantages and disadvantages to parents of having a yearly flu shot added to the other vaccination programs already delivered in schools. (If you ask a question about vaccinations during the discussion, we’ll ask you to defer those until after the focus group is finished.)

We will be tape recording our discussion, which will be about 1-2 hours long. Please turn all cell phones off, or to vibrate. Please try to remember only one person can speak at a time otherwise our typist won’t be able to understand the tape recording. (Please no side conversations for the same reason.) It’s important to us that all your voices are heard, and everyone has the opportunity to speak. Please don’t share anything that takes place in this room with someone who is not here – for confidentiality. Most importantly, there are no right/wrong answers, we want your perspectives, opinions and stories – we are here to find out your opinions and advice for us regarding adding a yearly flu vaccination to school programs for children in Alberta. You can share your own thoughts, agree or disagree with others, bring out a new point of view, or add onto or build upon each others’ thoughts that come out in our discussion today. Although we have prepared a few questions to lead the discussion, we’d like to have it be a pretty open, informal process so we can follow up on different directions the conversation might take. We want it to be a conversation in which everyone joins in.

Focus Group Questions

1. Can you tell me about your experiences with school-based vaccinations for your children?
2. Can you tell me your thoughts about adding a yearly flu vaccination to currently offered school based vaccination programs in Alberta?
   1. Where (for example, at what type of location) would you prefer your school-aged children (between the ages of 5-18) be vaccinated?
   2. Under what circumstances would you use a school-based flu vaccination program? What would make you want to use it? How do you make this decision?
3. What are the advantages to parents of school-based flu vaccination?
4. What are the disadvantages of a school-based flu vaccination program?
   1. What would prevent you from having your child vaccinated against the flu, at his or her school?
5. What problems or issues do you see might arise with adding a flu vaccination to the already existing school vaccination programs?
   1. What is your advice on how those issues could be handled?
6. What features should a school-based vaccination program include (i.e. what should it look like)?
7. Have we missed anything – is there a question you wished I’d asked?
8. Any other thoughts?

Question probes (for use as required):

1. Are there any other points of view?
2. Could you tell me more about that?
3. What was involved in that?
4. Could you give me an example of what you mean?
5. How did it begin?
6. Then what happened?
7. How did it feel?
8. What was the effect on the people involved, effect on others?
9. Route of vaccine administration (e.g. injection vs nose drops)?
10. Type of school (elementary, middle school, junior high or high school)?
